# Intact regulation of muscle expression and circulating levels of myokines in response to exercise in patients with type 2 diabetes

**DOI:** 10.14814/phy2.13723

**Published:** 2018-06-19

**Authors:** Rugivan Sabaratnam, Andreas J. T. Pedersen, Jonas M. Kristensen, Aase Handberg, Jørgen F. P. Wojtaszewski, Kurt Højlund

**Affiliations:** ^1^ Section of Molecular Diabetes & Metabolism Institute of Clinical Research Institute of Molecular Medicine University of Southern Denmark Odense C Denmark; ^2^ Department of Endocrinology Odense University Hospital Odense C Denmark; ^3^ Section of Molecular Physiology Department of Nutrition, Exercise and Sports University of Copenhagen Copenhagen Denmark; ^4^ Department of Clinical Biochemistry Aalborg University Hospital Aalborg Denmark; ^5^ Department of Clinical Medicine Aalborg University Aalborg Denmark

**Keywords:** Exercise resistance, myokines, skeletal muscle, T2D

## Abstract

Regular exercise plays an important role in the prevention and treatment of type 2 diabetes (T2D). The synthesis and secretion of myokines in response to contraction may contribute to the beneficial metabolic effects of exercise. However, some exercise‐induced responses may be attenuated in T2D. Here, we investigated whether the effect of acute exercise on selected myokines are impaired in T2D. Skeletal muscle biopsies and blood samples were obtained from 13 men with T2D and 14 weight‐matched, glucose‐tolerant men before, immediately after and 3‐h after acute exercise (60 min cycling) to examine muscle expression and plasma/serum levels of selected myokines. One‐hour of exercise increased muscle expression of *IL6, FGF21, ANGPTL4, CHI3L1, CTGF* and *CYR61,* of which *FGF21, ANGPTL4* and *CHI3L1* increased further 3‐h into recovery, whereas expression of *IL6, CYR61,* and *CTGF* returned to baseline levels. There was no immediate effect of exercise on *IL15* expression, but it decreased 3‐h into recovery. Plasma IL‐6 increased robustly, whereas circulating levels of FGF21, ANGPTL4, IL‐15, and CHI3L1 increased only modestly in response to exercise. All returned toward baseline levels 3‐h into recovery except for plasma ANGPTL4, which increased further. No significant differences in these responses to exercise were observed between the groups. Our results demonstrate that muscle expression and circulating levels of selected known and putative myokines were equally regulated by acute exercise in patients with T2D and weight‐matched controls. This suggests that the potential beneficial metabolic effects of these myokines are not impaired in patients with T2D.

## Introduction

Insulin resistance is an important hallmark in the pathogenesis of type 2 diabetes (T2D). Skeletal muscle insulin resistance is characterized by defective insulin signaling to glucose transport and glycogen synthesis, increased lipid accumulation, and mitochondrial dysfunction (Szendroedi et al. 2011; Hojlund 2014; Ritter et al. 2015). Physical inactivity increases the risk of developing T2D, while regular exercise can delay or prevent the onset of T2D (Helmrich et al. 1991; Hawley 2004; Madden 2013). Exercise is associated with numerous beneficial metabolic effects including improved maximal pulmonary oxygen uptake (VO_2max_), insulin sensitivity, and whole‐body glucose and lipid metabolism (Hawley 2004; Hawley et al. 2014). Accordingly, regular exercise activates various key metabolic pathways leading to enhanced abundance of proteins involved in insulin‐signaling, lipid and glucose metabolism, and mitochondrial oxidative metabolism (Egan and Zierath 2013). However, some studies have reported attenuated exercise‐mediated increases in insulin sensitivity, VO_2max_ and muscle expression of markers of mitochondrial oxidative metabolism in individuals with prediabetes and T2D indicating the existence of exercise resistance (De Filippis et al. 2008; Hernandez‐Alvarez et al. 2010; Stephens and Sparks 2015). Skeletal muscle is now widely accepted as an endocrine organ, which in response to contraction and other stimuli synthesizes and secretes peptides or proteins, termed myokines (Pedersen et al. 2007; Eckardt et al. 2014). Exercise‐induced release of myokines is of great interest, as myokines may play important roles in muscle physiology and metabolism, potentially mediating several beneficial metabolic effects by communicating with other tissues in an autocrine, paracrine, and endocrine manner (Pedersen and Febbraio 2012). However, to what extent production and release of myokines from skeletal muscle in response to acute exercise are impaired in patients with T2D remains to be established.

Recently, secretome analysis has led to the identification of hundreds of putative myokines induced by contraction in myotubes and human skeletal muscle (Catoire et al. 2014a; Hartwig et al. 2014; Pourteymour et al. 2017). However, the physiological function, regulation and whether these myokines are released from human skeletal muscle in vivo remain unclear. One of the most well‐established myokines is interleukin 6 (IL‐6), which is robustly induced and released from human skeletal muscle in response to acute exercise and exercise training (Steensberg et al. 2000; Keller et al. 2003; Christiansen et al. 2013; Langleite et al. 2016). The muscle expression and secretion of IL‐6 is dependent upon exercise intensity, duration and pre‐exercise glycogen content (Steensberg et al. 2000, 2001). Moreover, there is experimental evidence that IL‐6 may contribute to the insulin‐sensitizing and anti‐inflammatory effects of exercise, at least in healthy humans (Carey et al. 2006). However, IL‐6 infusion into patients with T2D did not improve insulin sensitivity (Harder‐Lauridsen et al. 2014). Interleukin‐15 (IL‐15) is another presumed myokine, which is believed to play a role in muscle growth, lipid and glucose metabolism, enhanced mitochondrial function and reduced fat mass (Duan et al. 2017). Acute exercise has been shown to increase the circulating levels of IL‐15 in healthy, lean, and obese individuals (Riechman et al. 2004; Tamura et al. 2011; Christiansen et al. 2013). Less consistently, studies have shown either increased (Louis et al. 2007) or no change (Nieman et al. 2004, 2003) in skeletal muscle expression of *IL15* after acute endurance exercise in healthy subjects.

Newly identified putative myokines include angiopoietin‐like 4 (ANGPTL4), chitinase 3‐like protein 1 (CHI3L1), and fibroblast growth factor 21 (FGF21), which are believed to play key roles in glucose and lipid metabolism, muscle growth and inflammation (Grootaert et al. 2012; Fisher and Maratos‐Flier 2016; Gorgens et al. 2016) as well as cysteine rich angiogenic inducer 61 (CYR61) and connective tissue growth factor (CTGF), which are involved in angiogenesis and remodeling of extracellular matrix (ECM) within skeletal muscle (Perbal 2004; Catoire et al. 2014a; Pourteymour et al. 2017). ANGPTL4 regulates lipid metabolism by inhibiting lipoprotein lipase (LPL) activity (Grootaert et al. 2012), while FGF21 is a member of the fibroblast growth factor superfamily, which among many proposed biological functions also has been described as a putative myokine and currently is being pursued as a therapeutic drug for obesity and T2D (Fisher and Maratos‐Flier 2016). CHI3L1 (or YKL‐40) is a glycoprotein, which has been reported to inhibit TNF*α*‐induced inflammation and insulin resistance in human skeletal muscle cells (Gorgens et al. 2014). Several studies in mice and healthy humans have shown increased muscle gene expression and circulating levels of CHI3L1 and ANGPTL4 in response to acute exercise (Kersten et al. 2009; Catoire et al. 2014a,b; Gorgens et al. 2016; Ingerslev et al. 2017). Some (Ozaki et al. 2005; Catoire et al. 2014b), but not all studies (Kim et al. 2013) in mice and healthy humans have observed increased muscle gene expression of FGF21 in response to acute exercise, whereas circulating levels of FGF21 are consistently reported to be increased in response to acute exercise (Kim et al. 2013; Hansen et al. 2016; Tanimura et al. 2016). For most novel putative myokines, it remains to be established whether skeletal muscle is the major origin of the exercise‐induced increase in circulating levels. Moreover, the circulating levels of IL‐6, CHI3L1, and FGF21 and less consistently ANGPTL4 have been reported to be elevated in patients with T2D and other insulin resistant conditions in the resting, fasting state (Duncan et al. 2003; Nielsen et al. 2008; Chavez et al. 2009; Cheng et al. 2011; Kruse et al. 2017; Tjeerdema et al. 2014; Xu et al. 2005). Together with the reported existence of exercise resistance in some cohorts of insulin resistant individuals, these observations suggest that the exercise‐mediated regulation of these known and putative myokines may be altered in patients with T2D. In line, an attenuated exercise‐induced increase in plasma FGF21 in patients with T2D was recently reported (Hansen et al. 2016).

The aim of the present study was to test the hypothesis that the exercise‐induced changes in muscle gene expression and circulating levels of selected known and putative myokines were attenuated in patients with T2D compared with weight‐matched controls, and whether the exercise‐induced changes in muscle expression and circulating levels of myokines were co‐regulated or correlated with body composition and metabolic characteristics.

## Materials and Methods

### Subjects

In the present study, we investigated skeletal muscle biopsies and blood samples from a larger study of the effects of insulin and acute exercise (Pedersen et al. 2015; Kjobsted et al. 2016; Kruse et al. 2016). In brief, 13 overweight/obese male type 2 diabetic patients and 14 obese, nondiabetic male control individuals group‐wise matched for age, BMI and self‐reported physical activity level (IPAQ) were recruited (Table 1). The type 2 diabetic patients were GAD65‐antibody negative and without signs of diabetic micro‐ or macrovascular complications. The control individuals had normal fasting glucose, normal glucose tolerance, and no family history of diabetes. The patients with T2D were treated either by diet alone (*n* = 4) or by diet in combination with metformin (*n* = 5) or metformin and sulfonylurea (*n* = 4). Seven days before each study day, all medications were withdrawn. Forty‐eight hours before each study day, the participants were instructed to avoid strenuous exercise. Informed consent was obtained from all participants before participation. The study was approved by The Regional Scientific Ethical Committees for Southern Denmark and was performed in accordance with the Helsinki Declaration.

**Table 1:** Clinical and metabolic characteristics of the participants

	Controls	Type 2 diabetes
*n*	14	13
Age (years)	54.7 ± 2.3	55.4 ± 2.0
BMI (kg m^−2^)	29.0 ± 0.9	29.7 ± 1.0
Fat free mass (kg)	69.1 ± 2.4	68.3 ± 2.0
Fat mass (kg)	24.5 ± 1.9	28.2 ± 2.4
Fasting plasma glucose (mmol L^−1^)	5.6 ± 0.1	10.0 ± 0.7***
HbA1c (%)	5.5 ± 0.1	7.0 ± 0.2***
Plasma triglycerides (mmol L^−1^)	1.5 ± 0.2	3.2 ± 1.5
IPAQ score	5248 ± 952	5558 ± 943
VO_2max_ (L min^−1^)	3.50 ± 0.2	3.22 ± 0.2
GDR, clamp (mg m^−2^ min^−1^)	349 ± 35	242 ± 35*
Diabetes duration (years)	–	3.5 ± 1.2

Data represent means ± SEM. *P < 0.05 and ***P < 0.001 versus controls.

### Experimental design

One week before the first experimental trial, all participants underwent blood screening tests, ECG and exercise tests to determine maximal workload (*W*_max_) capacity and maximal aerobic capacity (VO_2max_) as previously reported (Pedersen et al. 2015; Kjobsted et al. 2016; Kruse et al. 2016). All the participants underwent a baseline euglycemic‐hyperinsulinemic clamp and an exercise day separated by 4–8 weeks. After an overnight fast, the participants (baseline day) underwent a euglycemic–hyperinsulinemic clamp (4‐h insulin infusion, 40 mU·m^−2^·min^−1^) using labeled glucose infusates as reported in detail previously (Pedersen et al. 2015; Kjobsted et al. 2016). In patients with T2D, plasma glucose was allowed to decline to ~5.5  mmol L^−1^ before glucose infusion was started. Using Steele's nonsteady‐state equations adapted for labeled glucose infusates, we calculated total glucose disposal rates (GDR) for the last 30 min of the basal and the insulin‐stimulated states (Pedersen et al. 2015; Kjobsted et al. 2016). Distribution volume of glucose was taken as 200 mL kg^−1^ body weight and pool fraction as 0.65. After 4–8 weeks, the participants returned after an overnight fast for the exercise day. After they rested in the supine position for 30 min, the first muscle biopsy (basal) from *m*. *vastus lateralis* was taken. Next, the participants exercised on a cycle ergometer for 60 min at an intensity of 70% of VO_2max_. Immediately after completion of exercise, a second skeletal muscle biopsy (exercise) was obtained from the same leg 4–5 cm proximal to the first biopsy site. Before obtaining the third biopsy (recovery) from *m. vastus lateralis* of the other leg, the participants rested in bed for 3‐h. All the biopsies were obtained using a modified Bergström needle with suction under local anesthesia (10 mL lidocaine 2%). The muscle biopsies were immediately blotted free of visible blood and connective tissue and frozen in liquid nitrogen (Kjobsted et al. 2016; Kruse et al. 2016). Blood samples used for the study of myokines were taken immediately prior to the exercise bout, immediately after exercise and 3‐h into recovery. Plasma/serum samples were stored at −80°C until analysis.

### Blood analysis

Plasma CHI3L1 was measured by the Micro Vue YKL‐40 enzyme immunoassay from Quidel (San Diego, Ca) essentially as described by the manufacturer. According to the manufacturer, lower limit of quantitation is 15.6 ng mL^−1^ and the between‐run precision is 6–7% in a CHI3L1 range from 51.8 to 262.9 ng mL^−1^. Plasma IL‐15 was measured by the Human IL‐15 Quantikine sandwich ELISA from Biotechne (RnD Systems, Minneapolis, MN) essentially as described by the manufacturer. Minimal detectable concentration was less than 2 pg mL^−1^, and interassay CV 6.6–9.1% (range 9.9–184 pg mL^−1^). Plasma ANGPTL4 was measured by using the Human ANGPTL4 DuoSet Elisa from Biotechne (Minneapolis, MN) essentially as described by the manufacturer.

Plasma IL‐6 was measured by the human IL‐6 Quantikine HS (high sensitivity) ELISA from Biotechne (Minneapolis, MN) essentially as described by the manufacturer. According to the manufacturer, minimal detectable concentration is 0.039 pg mL^−1^, and interassay CV 6.5–9.6% (range 0.49–5.65 pg mL^−1^). Serum FGF‐21 was measured by the Human FGF‐21 ELISA assay from BioVendor (Brno, Czech Republic) essentially as described by the manufacturer. According to the manufacturer, limit of detection is 7 pg mL^−1^, and interassay CV 3.1–3.5% (range 250–320 pg mL^−1^).

### RNA extraction, reverse transcription, and quantitative real‐time PCR

Total RNA was extracted from 50–75 mg of muscle as previously described (Kristensen et al. 2014). cDNA synthesis was performed using High Capacity cDNA Reverse Transcription kit (Life Technologies/ Applied Biosystems, Foster City, CA) according to manufacturer′s instructions. Quantitative real‐time PCR was performed on a 7900HT Fast Real‐Time PCR System (Applied Biosystems/Life Technologies, Foster City, CA) using TaqMan Custom Arrays (Life Technologies/Applied Biosystems, Foster City, CA) according to the manufacturer′s instructions. Primers used for quantitative real‐time PCR analysis are listed in Table S1. All samples were run in triplicates. Gene expression data was analysed using qBase+ Biogazelle software (Zwijnaarde, Belgium)( Vandesompele et al. 2002; Hellemans et al. 2007) and normalized to the geometric mean of two reference genes, peptidylprolyl isomerase A (*PPIA*) and beta‐2‐microglobulin (*B2M*).

### Statistical analyses

Statistical analyses were performed using SigmaPlot 13.0 software (Systat Software, San Jose, CA). Two‐way ANOVA with repeated measures, followed by Student‐Newman‐Keul post hoc analysis were performed when comparisons were made among groups. When no interaction between groups was observed, the *P*‐values report a main effect. Correlation analyses were performed using Spearman rank correlation coefficient. Data are expressed as means ± SEM. The significance level was accepted at *P* < 0.05.

## Results

### Clinical characteristics

As reported before (Pedersen et al. 2015; Kjobsted et al. 2016; Kruse et al. 2016), HbA1c and plasma glucose levels were significantly elevated in patients with T2D compared with weight‐matched controls, whereas no significant differences in body composition, VO_2max_ or W_max_ were observed. Insulin sensitivity measured as insulin‐stimulated GDR was reduced (~30%) in the diabetic group compared to the control group (Table 1) (Pedersen et al. 2015; Kjobsted et al. 2016; Kruse et al. 2016).

### Muscle expression and plasma levels of IL‐6 and IL‐15

**Figure 1:**
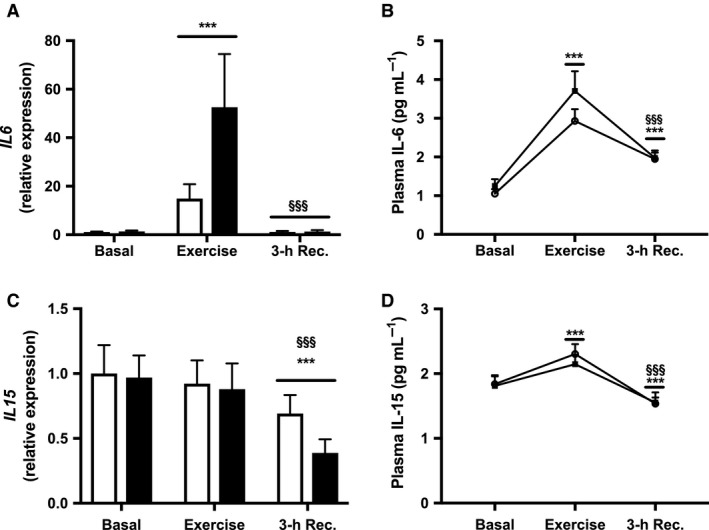
Muscle expression and plasma levels of IL‐6 and IL‐15. (A) *IL6* mRNA, (B) Plasma IL‐6, (C) *IL15* mRNA and (D) Plasma IL‐15 in patients with T2D (black bars, ∎) and healthy controls (white bars, ○) before (Basal) and immediately after 60 min of exercise at 70% VO_2max_ (Exercise) and 3‐h into recovery (3‐h Rec.). Values are means ± SEM. mRNA levels were measured in *n *= 13 controls and *n *= 10 patients with T2D. ***P < 0.001 versus Basal (main effect), and ^§§§^ *P* < 0.001 versus Exercise (main effect).

Acute exercise robustly increased muscle *IL6* mRNA level (*P* < 0.001) in weight‐matched controls and patients with T2D compared with the basal levels, whereas 3‐h into recovery, expression of *IL6* returned (main effect, *P* < 0.001) to basal levels in both groups compared with immediately after exercise (Fig. 1A). Plasma IL‐6 increased (*P* < 0.001) ~200% in both groups immediately after exercise, but then decreased toward basal levels 3‐h into recovery (*P* < 0.001), however, still being increased (~2‐fold) (*P* < 0.001) compared to basal levels (Fig. 1B). Muscle expression of *IL15* did not change in response to acute exercise, but decreased slightly 3‐h into recovery in both groups compared with basal levels (*P* < 0.001) and immediately after exercise (*P* < 0.001) (Fig. 1C). In contrast, plasma IL‐15 increased (*P* < 0.001) modestly (19–25%) in both groups immediately after exercise. Plasma IL‐15 decreased (*P* < 0.001) 3‐h into recovery in both groups compared with both basal values and immediately after exercise (Fig. 1D). No significant differences in muscle expression or plasma levels of IL‐6 or IL‐15 in response to exercise or recovery were observed between patients with T2D and weight‐matched controls (Fig. 1A–D).

### Muscle expression and serum levels of FGF21

**Figure 2:**
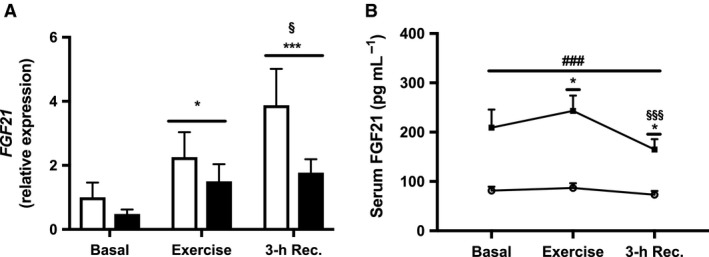
Muscle expression and serum levels of FGF21. (A) *FGF21* mRNA and (B) Plasma FGF‐21in patients with T2D (black bars, ∎) and healthy controls (white bars, ○) before (Basal) and immediately after 60 min of exercise at 70% VO_2max_ (Exercise) and 3‐h into recovery (3‐h Rec.). Values are means ± SEM. mRNA levels were measured in *n *= 13 controls and *n *= 10 patients with T2D. *P < 0.05 and ***P < 0.001 versus basal (main effect), ^§^ *P* < 0.05 and ^§§§^ *P* < 0.001 versus exercise (main effect), and ^###^ *P* < 0.001 versus healthy controls (main effect).

Muscle expression of *FGF21* increased (*P* = 0.019) in response to acute exercise in both groups compared with basal levels, and increased further 3‐h into recovery compared with after exercise (*P* < 0.001) (Fig. 2A). Serum FGF21 increased (*P* = 0.042) modestly (6–16%) in response to exercise in both groups compared to basal levels, but then declined (10–21%) to levels lower than both after exercise (*P* < 0.001) and in the basal state (*P* = 0.021) (Fig. 2B). Serum FGF21 was higher in patients with T2D throughout the exercise study (*P* < 0.001), but otherwise no significant differences in expression of FGF21 or changes in muscle expression or serum levels of FGF21 in response to exercise or recovery were observed between patients with T2D and weight‐matched controls (Fig. 2).

### Muscle expression and plasma levels of ANGPTL4 and CHI3L1

**Figure 3:**
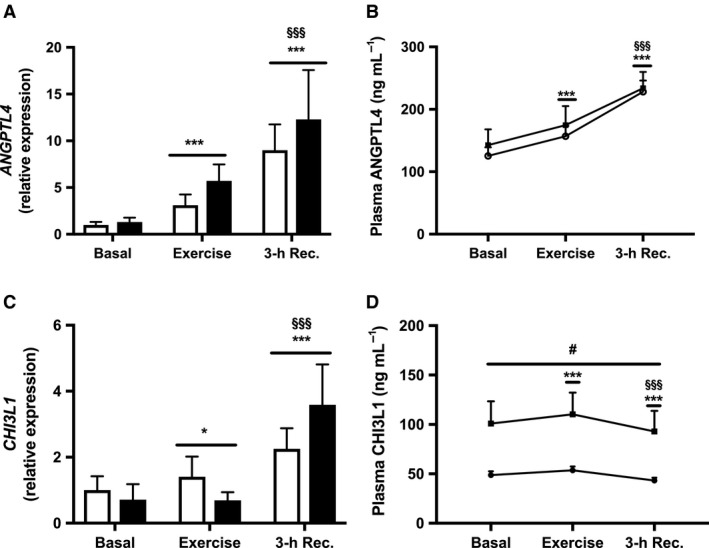
Muscle expression and plasma levels of ANGPTL4 and CHI3L1. (A) *ANGPTL4* mRNA, (B) Plasma ANGPTL4, (C) *CHI3L1* mRNA and (D) Plasma CHI3L1 in patients with T2D (black bars, ∎) and healthy controls (white bars, ○) before (Basal) and immediately after 60 min of exercise at 70% VO_2max_ (Exercise) and after 3‐h into recovery (3‐h Rec.). Values are means ± SEM. mRNA levels were measured in *n *= 13 controls and *n *= 11 patients with T2D. *P < 0.05 and ***P < 0.001 versus basal (main effect), ^§§§^ *P* < 0.001 versus exercise (main effect), and ^#^ *P* < 0.05 versus healthy controls (main effect).

Muscle expression of *ANGPTL4* (*P* < 0.001) and *CHI3L1* (*P* < 0.05) increased immediately after exercise in both groups compared with basal levels, and both expression of *ANGPTL4* and *CHI3L1* increased further 3‐h into recovery compared with after exercise (both *P* < 0.001) (Fig. 3A and C). In line, plasma ANGPTL4 increased (*P* < 0.001) in response to acute exercise in both groups compared with basal levels, and continued to increase 3‐h into recovery compared with after exercise (*P* < 0.001) (Fig. 3B). In contrast, plasma CHI3L1 increased (*P* < 0.001) only modestly (~9%) in response to acute exercise in both groups, and then 3‐h into recovery declined to levels lower than both after exercise (*P* < 0.001) and in the basal state (*P* < 0.001) (Fig. 3D). Plasma CHI3L1 was higher in patients with T2D throughout the exercise study (*P* < 0.05) (Fig. 3D), but otherwise no significant differences in plasma ANGPTL4, muscle expression of ANGPTL4 or CHI3L1, or exercise‐induced changes in expression or plasma levels of ANGPTL4 or CHI3L1 were observed between patients with T2D and weight‐matched controls (Fig. 3A–D).

### Muscle expression of *CYR61* and *CTGF*

**Figure 4:**
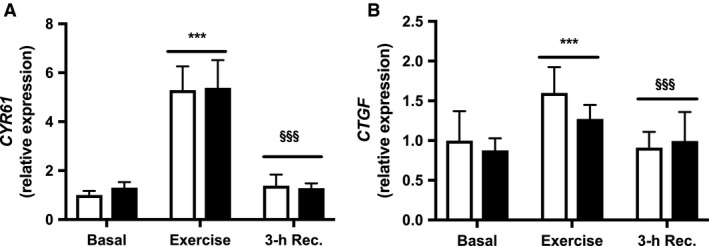
Muscle expression of *CYR61* and *CTGF*. (A) *CYR61* mRNA and (B) *CTGF* mRNA in patients with T2D (black bars) and healthy controls (white bars) before (Basal) and immediately after 60 min of exercise at 70% VO_2max_ (Exercise) and after 3‐h into recovery (3‐h Rec.). Values are means ± SEM. mRNA levels were measured in *n *= 13 controls and *n *= 13 patients with T2D. ***P < 0.001 versus basal (main effect), and ^§§§^ *P* < 0.001 versus exercise (main effect).

The muscle transcript levels of *CYR61* and *CTGF* did not differ between patients with T2D and weight‐matched controls either before, after an acute bout of exercise or 3‐h into recovery (Fig. 4). However, acute exercise robustly increased the expression of *CYR61* (*P* < 0.001) in both groups compared with basal levels, whereas muscle expression of *CTGF* was only modestly increased (*P* < 0.001) in response to exercise in both groups. Both the expression of *CYR61* and *CTGF* returned to basal levels (*P* < 0.001) compared with after exercise. There were no significant differences in the exercise‐induced changes in expression of *CYR61* and *CTGF* between patients with T2D and weight‐matched controls.

### Correlation analyses

Correlation analysis was performed to examine the potential relationship between muscle expression and circulating levels of myokines in response to acute exercise and the influence of body composition and metabolic characteristics in the total study cohort (*n *= 27). Interestingly, we found no significant correlations between the muscle transcript levels and the corresponding plasma/serum levels of IL‐6, IL‐15, FGF21, or CHI3L1 either before, immediately after exercise or 3‐h into recovery, except for a correlation between the expression of *ANGPTL4* and plasma levels of ANGPTL4 3‐h into recovery (*r *= 0.436, *P* = 0.02). Importantly, we found no significant correlation between the exercise‐induced changes in muscle mRNA levels and the corresponding changes in plasma/serum levels of IL‐6, IL‐15, FGF21, ANGPTL4, or CHI3L1.

BMI and fat mass correlated inversely with the exercise‐induced muscle expression of *CHI3L1* (*r *= −0.59, *P* < 0.01), *CYR61* (*r *= −0.44, *P* = 0.02), and *CTGF* (*r *= −0.60, *P* < 0.01). Moreover, BMI correlated with plasma IL‐6 (*r *= 0.46, *P* = 0.02) and fat mass correlated with basal plasma IL‐6 (*r *= 0.48, *P* = 0.01) and serum FGF21 throughout the study (*r *= 0.40–0.41, *P* < 0.05). Fasting plasma glucose and HbA1c correlated with serum FGF21 (*r *= 0.64–0.75, all *P* < 0.01) and plasma CHI3L1 (*r *= 0.41–0.52, all *P* < 0.05) before, immediately after exercise and 3‐h into recovery. Accordingly, insulin sensitivity measured as insulin‐stimulated Rd on a separate day correlated inversely with serum FGF21 at all time points on the exercise day (*r *= 0.40–0.49, all *P* < 0.05) and inversely with plasma CHI3L1 (*r *= 0.40–0.42, all *P* < 0.05) immediately after exercise and 3‐h into recovery. Plasma free fatty acids (FFA) before exercise correlated positively with expression of *ANGPTL4* (*r *= 0.59, *P* < 0.01) before exercise and with the exercise‐induced expression of *FGF21* (*r *= 0.44, *P* = 0.03) and *ANGPTL4* (*r *= 0.47, *P* = 0.02), but inversely with the exercise‐induced expression of *IL15* (*r *= −0.51, *P* < 0.01). Plasma FFA 3‐h into recovery correlated positively with muscle expression of *ANGPTL4* (*r *= 0.50, *P* = 0.02) at this time.

## Discussion

In this study, we examined the effect of a moderate‐intensity exercise stimulus on the muscle mRNA levels and circulating levels of selected myokines in patients with T2D and weight‐matched, glucose tolerant men. The novel findings of this study are that patients with T2D show an intact regulation of muscle gene expression and circulating levels of a panel of both well‐established (IL‐6) and novel putative myokines (IL‐15, FGF21, ANGPTL4, CHI3L1, CYR61 and CTGF) in response to acute exercise. Thus, the potential beneficial metabolic effects of the exercise‐mediated synthesis and release of these myokines do not seem to be impaired in patients with T2D. However, the dissociated pattern of changes in muscle expression and circulating levels of some of these myokines (IL‐15, FGF21, ANGPTL4, CHI3L1) as well as the lack of correlation between the changes in each of these myokines after exercise and 3‐h into recovery, emphasizes that further studies are needed to establish the source and the role of at least some of these exercise‐induced myokines.

Several studies have reported that muscle mRNA and circulating levels of IL‐6 are increased in response to acute exercise in healthy, lean and obese individuals (Steensberg et al. 2000, 2001; Keller et al. 2003; Christiansen et al. 2013). Similarly, studies have demonstrated that acute exercise increases circulating levels of IL‐15 in humans (Riechman et al. 2004; Tamura et al. 2011; Christiansen et al. 2013), whereas the effect of acute exercise on muscle expression of *IL15* in humans is unclear (Nieman et al. 2004, 2003; Louis et al. 2007). Consistently, we here report an intact and robust increase in muscle expression and circulating levels of IL‐6 immediately after exercise in both patients with T2D and weight‐matched controls. Thus, the most well‐established of the examined myokines responded to acute exercise as expected providing proof of principle with respect to study design. As a novel finding, we demonstrate for the first time an exercise‐induced increase in circulating levels of IL‐15 in patients with T2D similar to the increase observed in weight‐matched controls. However, no increase in muscle expression of *IL15* was observed in either group immediately after exercise, whereas muscle expression of *IL15* decreased 3‐h into recovery. A previous study reported increased muscle mRNA levels of *IL15* 24‐h after heavy bout of resistance exercise (Nielsen et al. 2007), emphasizing that the regulation of *IL15* mRNA levels and serum Il‐15 may depend on the exercise type, the athletic level of the individuals and timing of the muscle and blood samples in different studies (Pistilli and Quinn 2013). The dissociated pattern of changes and the lack of correlation between changes in muscle mRNA and circulating levels of IL‐15, suggest that muscle might not be the major tissue responsible for elevated circulating levels of IL‐15 in response to acute exercise in humans. Thus, further studies are needed to establish the putative role of IL‐15 as a myokine in humans.

FGF21 is an important regulator of glucose and lipid metabolism and several studies have reported that muscle expression and circulating FGF21 levels are increased by acute exercise in mice and healthy humans (Fisher and Maratos‐Flier 2016; Tanimura et al. 2016), although in one study of mice the exercise‐induced increase in plasma FGF21 was not accompanied by increased muscle expression of *FGF21* (Kim et al. 2013). It was recently reported that the exercise‐induced (60 min at 50% of VO_2max_) increase in plasma FGF21 was absent in a small cohort of patients with T2D, when evaluated as the fold‐change relative to baseline levels (Hansen et al. 2016). However, in neither non‐obese patients with T2D nor obese/overweight controls, exercise changed plasma FGF21 levels when analysed in absolute concentrations (Hansen et al. 2016). In contrast, we investigated a larger cohort of patients with T2D and weight‐matched controls and were able to demonstrate both increased muscle expression and elevated circulating levels of FGF21 in humans in response to acute exercise. Moreover, we for the first time demonstrate that both these responses to acute exercise are intact in patients with T2D. Nevertheless, the exercise‐induced changes in muscle *FGF21* mRNA expression were dissociated from the changes in serum FGF21 as evidenced by a continued increase in muscle *FGF21* expression despite a marked reduction in serum FGF21 levels 3‐h into recovery. This, together with the lack of correlation between exercise‐induced changes in muscle expression and serum levels of FGF21, indicates that tissues other than skeletal muscle are responsible for the regulation of circulating FGF21 in response to exercise. This is supported by recent studies of mice and humans, which have provided evidence that the liver is a major contributor to the exercise‐induced increase in circulating FGF21 (Kim et al. 2013; Hansen et al. 2016; Tanimura et al. 2016). The role of FGF21 as a myokine was first suggested by experimental work showing increased muscle expression and release of FGF21 in Akt1 overexpressing mice (Izumiya et al. 2008). This was supported by studies showing increased muscle expression and serum FGF21 levels in response to physiological concentrations of insulin in humans (Hojman et al. 2009; Kruse et al. 2017). However, as reported previously (Kjobsted et al. 2016), serum insulin levels declined ~50% immediately after exercise in our study, supporting that the increase in circulating FGF21 in response to acute exercise does not originate from skeletal muscle but rather from the liver, where it seems to be mediated by an elevated glucagon‐to‐insulin ratio (Hansen et al. 2015a).

ANGPTL4 is a potent inhibitor of LPL activity and regulator of lipid metabolism (Grootaert et al. 2012). In agreement with previous exercise studies in nondiabetic individuals (Kersten et al. 2009; Catoire et al. 2014a,b; Norheim et al. 2014; Ingerslev et al. 2017), we are the first to demonstrate an intact upregulation of muscle *ANGPTL4* mRNA and increase in circulating ANGPTL4 in response to acute exercise and further 3‐h into recovery in patients with T2D. Experimental work in mice and humans have provided evidence that exercise markedly induces expression of *ANGPTL4* in both adipose tissue and liver, and that ANGPTL4 is mainly released from the liver in response to exercise (Norheim et al. 2014; Ingerslev et al. 2017). These data suggest that other tissues may contribute more than skeletal muscle to the exercise‐induced increase in circulating ANGPTL4. Although we observed a similar pattern of changes in muscle expression and circulating levels of ANGPTL4, we found no correlation between these changes, which supports the hypothesis that muscle is not the major source of plasma ANGPTL4 in response to exercise (Catoire et al. 2014a). Several studies have indicated that increased circulating ANGPTL4 levels are mediated by elevation in plasma FFA (Kersten et al. 2009; Catoire et al. 2014b; Norheim et al. 2014). We have previously reported that exercise caused increased plasma FFA, which was further elevated 3‐h into recovery in this study cohort (Pedersen et al. 2015). This suggests that increased plasma FFA mediated the increased muscle expression and circulating levels of ANGPTL4. In agreement, correlation analyses showed that muscle expression of *ANGPTL4* was positively associated with FFA before exercise and 3‐h into recovery, although not immediately after exercise. Nevertheless, further studies are necessary to understand the regulation and role of changes in circulating ANGPTL4 during exercise.

In cultured human skeletal muscle cells, CHI3L1 has been shown to protect against TNF‐*α* mediated inflammation and insulin resistance and to promote muscle growth and repair (Gorgens et al. 2014). In line with a previous study reporting an exercise‐induced increase in muscle expression and circulating levels of CHI3L1 in non‐diabetic individuals (Gorgens et al. 2016), we here, for the first time, demonstrate an intact exercise‐induced increase in muscle expression of CHI3L1, in particular 3‐h into recovery, in patients with T2D, as well as an intact but only modest and transient increase in circulating CHI3L1. The dissociated pattern of changes and the lack of correlation between changes in muscle mRNA and circulating levels of CHI3L1 suggest that circulating CHI3L1 is not regulated by changes in muscle expression of this presumed myokine. IL‐6 or TNF‐*α* might regulate CHI3L1 as suggested by Gorgens et al. (2014). However, we did not identify any association between either muscle mRNA and circulating levels of CHI3L1 and IL‐6 at any time‐points or when comparing changes induced by exercise.

In the present study, we observed elevated circulating levels of FGF21 and CHI3L1 in patients with T2D not only in the basal, resting state but also during the exercise study. Moreover, circulating FGF21 and CHI3L1 correlated positively with fasting plasma glucose and HbA1c, but inversely with insulin sensitivity in the total study cohort. These findings are consistent with several other studies, which has demonstrated elevated circulating levels of FGF21 and CHI3L1 in patients T2D (Nielsen et al. 2008; Chavez et al. 2009; Cheng et al. 2011; Kruse et al. 2017). However, in contrast to our hypothesis, these alterations did not attenuate the exercise‐induced regulation of these myokines in the muscle or the circulation. Circulating levels of ANGPTL4 has been reported to be both elevated (Tjeerdema et al. 2014) and reduced in patients with T2D (Xu et al. 2005), which could be explained by the interindividual variability in ANGPTL4 levels (Kersten et al. 2009). However, we observed no differences in plasma ANGPTL4 levels between patients with T2D and weight‐matched individuals in our study. Interestingly, we showed that plasma IL‐6 correlates strongly with BMI and fat mass, suggesting that IL‐6 levels are strongly associated with overweight/obesity, which is supported by other studies (Khaodhiar et al. 2004).

ECM associated proteins such as CYR61 and CTGF are important players in regulating endothelial function and angiogenesis (Perbal 2004). Recently, secretome analysis of muscle transcripts that changed in response acute exercise, suggested CYR61 and CTGF as exercise‐induced putative myokines that are likely to be secreted from human skeletal muscle (Catoire et al. 2014a). Moreover, microarray analysis showed a similar exercise‐induced increase in muscle *CTGF* expression in non‐obese patients with T2D and overweight controls (Hansen et al. 2015b). In accordance with these findings (Catoire et al. 2014a; Hansen et al. 2015b), we observed an immediate and equal increase in muscle gene expression of *CYR61* and *CTGF* in response to exercise in patients with T2D and weight‐matched groups. However, plasma levels of CYR61 did not change in response to acute exercise in healthy humans (Catoire et al. 2014a), suggesting that these putative myokines may act only locally on muscle cells.

The limitations of our study include a small study cohort, the lack of a lean, healthy group to exclude the possibility that obesity/overweight itself causes exercise resistance, as well as lack of inclusion of women in both study groups in order to rule out gender‐specific effects of exercise on the examined myokines. Moreover, access to adipose tissue biopsies as well as the application of measurements of the arterial‐to‐venous difference over the leg and the hepato‐splanchnic bed would have been helpful to determine the origin of the examined myokines in response to exercise (Hansen et al. 2016; Ingerslev et al. 2017).

In summary, our study provides novel evidence that the effects of an acute bout of exercise on muscle gene expression and circulating levels of selected well‐established and novel putative myokines are intact in patients with T2D compared with weight‐matched controls. Thus, the potential beneficial metabolic effect of the exercise‐induced elevation in the circulating levels of these putative myokines does not seem to be attenuated in patients with T2D. However, the dissociation in patterns of exercise‐induced changes in muscle expression and circulating levels of these myokines indicate that other organs may contribute more than muscle to the exercise‐induced changes in circulating levels of these presumed myokines, and that the contraction‐mediated increase in muscle expression of at least some of these myokines may play a more local role, regulating muscle adaptation to exercise in an autocrine manner.

## Conflicts of Interest

The authors have nothing to disclose.

## Data Accessibility

## Supporting information




**Table S1.** qRT‐PCR primer and probe information of genes studied including reference genes. TaqMan assay ID (Life Technologies/Applied Biosystems, Foster City, CA).
