# Supplementary material for: Intact regulation of muscle expression and circulating levels of myokines in response to exercise in patients with type 2 diabetes
Source: Physiol Rep. 2018 Jun 19;6(12):e13723. doi: 10.14814/phy2.13723 (PMC6009776; doi:10.14814/phy2.13723)
Supplement: Supplementary file 1 — Table S1. qRT‐PCR primer and probe information of genes studied including reference genes. TaqMan assay ID (Life Technologies/Applied Biosystems, Foster City, CA). [file PHY2-6-e13723-s001.docx]

**Supplemental table 1**

**qRT-PCR primer and probe information of genes studied. TaqMan assay ID (Life Technologies/Applied Biosystems, Foster City, CA, USA).**

| **Gene symbol** | **TaqMan Assay ID** |
| --- | --- |
| Reference genes | |
| *B2M* | Hs00984230_m1 |
| *PPIA* | Hs04194521_s1 |
| Target genes | |
| *ANGPTL4* | Hs01101127_m1 |
| *CHI3L1* | Hs01072228_m1 |
| *CTGF* | Hs00170014_m1 |
| *CYR61* | Hs00155479_m1 |
| *FGF21* | Hs00173927_m1 |
| *IL15* | Hs01003716_m1 |
| *IL6* | Hs00174131_m1 |
